# Sevoflurane postconditioning alleviates hypoxia-reoxygenation injury of cardiomyocytes by promoting mitochondrial autophagy through the HIF-1/BNIP3 signaling pathway

**DOI:** 10.7717/peerj.7165

**Published:** 2019-06-24

**Authors:** Long Yang, Jianjiang Wu, Peng Xie, Jin Yu, Xin Li, Jiang Wang, Hong Zheng

**Affiliations:** 1 Department of Anesthesiology, The First Affiliated Hospital of Xinjiang Medical University, Urumqi, Xinjiang, China; 2 Department of Anesthesiology, Zunyi Medical College, Guizhou Key Laboratory of Anesthesia and Organ Protection, Zunyi, Guizhou, China; 3 Chongqing Health Center for Women and Children, Department of Anesthesiology, Chongqing, Chongqing, China

**Keywords:** Hypoxia-reoxygenation injury, Sevoflurane postconditioning, Myocardial protection, Autophagy

## Abstract

**Background:**

Sevoflurane postconditioning (SpostC) can alleviate hypoxia-reoxygenation injury of cardiomyocytes; however, the specific mechanism remains unclear. This study aimed to investigate whether SpostC promotes mitochondrial autophagy through the hypoxia-inducible factor-1 (HIF-1)/BCL2/adenovirus E1B 19-kDa-interacting protein 3 (BNIP3) signaling pathway to attenuate hypoxia-reoxygenation injury in cardiomyocytes.

**Methods:**

The H9C2 cardiomyocyte hypoxia/reoxygenation model was established and treated with 2.4% sevoflurane at the beginning of reoxygenation. Cell damage was determined by measuring cell viability, lactate dehydrogenase activity, and apoptosis. Mitochondrial ultrastructural and autophagosomes were observed by transmission electron microscope. Western blotting was used to examine the expression of HIF-1, BNIP3, and Beclin-1 proteins. The effects of BNIP3 on promoting autophagy were determined using interfering RNA technology to silence BNIP3.

**Results:**

Hypoxia-reoxygenation injury led to accumulation of autophagosomes in cardiomyocytes, and cell viability was significantly reduced, which seriously damaged cells. Sevoflurane postconditioning could upregulate HIF-1α and BNIP3 protein expression, promote autophagosome clearance, and reduce cell damage. However, these protective effects were inhibited by 2-methoxyestradiol or sinBNIP3.

**Conclusion:**

Sevoflurane postconditioning can alleviate hypoxia-reoxygenation injury in cardiomyocytes, and this effect may be achieved by promoting mitochondrial autophagy through the HIF-1/BNIP3 signaling pathway.

## Introduction

Oxidative phosphorylation generates energy in mitochondria in cardiomyocytes. Functionally intact mitochondria are important for the heart to maintain physiological activities. However, myocardial ischemia-reperfusion (I/R) injury can lead to mitochondrial damage, and damaged mitochondria can produce a large amount of reactive oxygen species (ROS) to further attack normal mitochondria, leading to cardiomyocyte death (Zorov, Juhaszova & Sollott, 2014). Therefore, it is critical to remove dysfunctional mitochondria in a timely manner to avoid cardiomyocyte damage. Mitochondrial autophagy (Lemasters, 2005) is a defensive metabolic process that cells use to adapt to hypoxia that has been identified in recent years. It can selectively remove damaged mitochondria that are aged or excessively producing ROS through autophagy and promote mitochondrial renewal and recycling to ensure stable mitochondrial function and promote cell survival (Mizushima & Komatsu, 2011). At present, this adaptive metabolic mechanism has attracted much attention in the field of hypoxic stress.

Studies have shown that mitochondrial autophagy is the only identified mitochondrial renewal mechanism and is closely related to hypoxia (Gui, Liu & Lv, 2016). Hypoxia-induced mitochondrial autophagy is a protective mechanism of the cell, and activation of mitochondrial autophagy is dependent on autophagy-related proteins. BCL2/adenovirus E1B 19-kDa-interacting protein 3 (BNIP3) is a mitochondrial autophagy receptor that is closely related to hypoxia (Bellot et al., 2009). It is considered to be an important signaling molecule for hypoxia-induced mitochondrial autophagy and is a member of the BH3 domain only subfamily of the Bcl-2 family that is located in the mitochondrial outer membrane (Semenza, 2011). The expression level of BNIP3 is low under normal physiological conditions but increases sharply under conditions of ischemia and hypoxia (Wang, Ma & Qi, 2012). Studies have found that during myocardial I/R injury, BNIP3 can induce mitochondrial permeability changes, aggregation of autophagosomes and consumption of lysosomes and promote the occurrence of mitochondrial autophagy (Ma et al., 2012a). Under hypoxic conditions, the transcription of BNIP3 is activated by the regulation of hypoxia-inducible factor-1α (HIF-1α). Bellot et al., (2009) demonstrated a variety of normal cells that could activate downstream BNIP3 gene transcription through HIF-1 when they were exposed to hypoxia to promote mitochondrial autophagy, thereby removing damaged mitochondria.

Sevoflurane is an inhaled anesthetic that is widely used in clinical and basic research. It has unique pharmacological characteristics such as stable induction and rapid recovery. Many studies (Cao et al., 2015; Qiao et al., 2018; Wu et al., 2017) have confirmed that sevoflurane postconditioning (SpostC) can effectively alleviate I/R injury of healthy cardiomyocytes. Our previous studies demonstrated that SpostC could counteract myocardial I/R injury by upregulating HIF-1α expression (Yang et al., 2016). However, the specific molecular mechanisms have not been elucidated. Recent studies have found that autophagy plays a key role in SpostC myocardial protection (Yu et al., 2015; Zhang et al., 2014). Therefore, is the myocardial protection of SpostC associated with HIF-1/BNIP3-regulated autophagy?

This study used an H9C2 cardiomyocyte hypoxia-reoxygenation model to explore the possible molecular mechanism of the effects of SpostC on reducing hypoxia-reoxygenation injury and the HIF-1/BNIP3 signaling pathway from the perspective of mitochondrial autophagy to provide theoretical evidence for the myocardial protection mechanism of SpostC.

## Materials and Methods

### Cell culture and processing

The H9C2 rat embryonic cardiomyocyte cell line was obtained from Kaiji Biological Co., Ltd., China. The cell culture conditions consisted of DMEM (high sugar) medium + 10% FBS at 37 °C, 5% CO_2_, and saturated humidity. H9C2 cells with good growth at 90% confluency were used to prepare a 5 × 10^4^ cell/ml single-cell suspension using complete medium. Cells were inoculated in 96-well plates and incubated for 24 h at 37 °C in a 5% CO_2_ incubator. When the cells grew to 80% confluency, the supernatant was discarded, the adherent cells were washed twice with PBS, and serum-free DMEM (low sugar) medium was added. The plates were placed in a saturated tri-gas incubator at 37 °C with 95% N_2_ and 5% CO_2_ for 3 h. After culturing under hypoxic conditions, the cells were removed, the supernatant was discarded, fresh serum-free DMEM (low sugar) medium was added, and cells were re-oxygenated in a CO_2_ incubator for 3 h.

### Sevoflurane postconditioning (SpostC) of H9C2 cardiomyocyte

According to the previous study (Obal et al., 2001; Yu et al., 2016), a Vapor 2000 sevoflurane vaporizer (Drager, Lubeck, Germany) was used to apply a gas mixture containing 97.6% O_2_ and 2.4% sevoflurane. Briefly, an in-line sevoflurane vaporizer fed a supply of gas mixture containing 97.6% O_2_ and 2.4% sevoflurane for at least 10 min until the desired sevoflurane concentration (2.4%) was achieved. Concentrations of sevoflurane and O_2_ were monitored using an anesthetic analyzer (Drager Vamous, Lubeck, Germany) in the outlet. The gas flow rate was two l/min. After the cells were treated for 15 min, they were taken out immediately and incubated in a 5% CO_2_ cell incubator for 165 min at 37 °C.

Hypoxia-inducible factor-1α inhibitor 2-methoxyestradiol (2ME2) treatment steps: H9C2 cells were cultured in medium (containing 2 μM 2ME2 dissolved in 0.02% DMSO) for 15 min at the beginning of reoxygenation after 3 h of hypoxia, and then cultured in medium without 2ME2 in a 5% CO_2_ cell incubator for 165 min. Other groups were only cultured in fresh medium for the corresponding times.

### Experimental grouping

**Figure 1:**
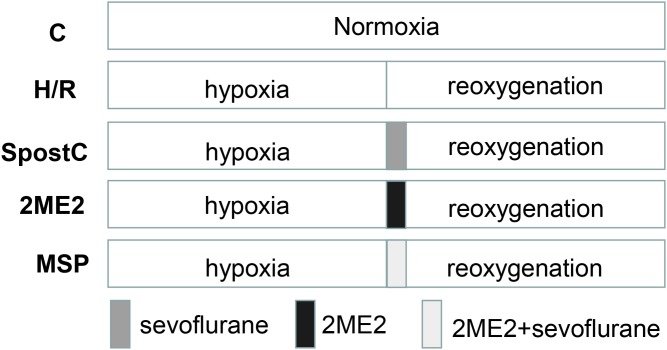
Experimental schemes of each group. H9C2 cells were randomly divided into control (C) group, hypoxia/reoxygenation (H/R) group, sevoflurane postconditioning (SPostC) group, HIF-1α inhibitor 2-methoxyestradiol (2ME2) group and 2ME2 + SpostC (MSP) group.

The experiment was divided into five groups: the control group (C): H9C2 cells were incubated for 6 h in normoxia. Hypoxia-reoxygenation group (H/R): hypoxia for 3 h and reoxygenation for 3 h. Sevoflurane postconditioning group (SpostC): H9C2 cells were exposed to 2.4% sevoflurane for 15 min at the beginning of reoxygenation after 3 h of hypoxia, and then incubated in a 5% CO_2_ cell incubator for 165 min. Hypoxia-inducible factor-1α inhibitor 2ME2 group: H9C2 cells were cultured in medium (containing 2 μM 2ME2) for 15 min at the beginning of reoxygenation after 3 h of hypoxia, and then cultured in medium without 2ME2 in a 5% CO_2_ cell incubator for 165 min. 2ME2 + SpostC group (MSP): H9C2 cells were cultured in medium (containing 2 μM 2ME2) in a hypoxia-reoxygenation box filled with 2.4% sevoflurane for 15 min at the beginning of reoxygenation after 3 h of hypoxia, and then cultured in medium without 2ME2 in a 5% CO_2_ cell incubator for 165 min (Fig. 1).

### RNA interference and gene transfection

siRNA oligonucleotides were synthesized by GenePharma Co., Ltd. (Shanghai, China), and the siRNA gene sequences were as follows: BNIP3-FsiRNA: 5′-CCU GGG UAG AAC UGC ACU UTT-3′, BNIP3-RsiRNA: 5′-AAG UGC AGU UCU ACC CAG GTT-3′, NC-FsiRNA: 5′-UUC UCC GAA CGU GUC ACG UTT-3′, NC-RsiRNA: 5′-ACG UGA CAC GUU CGG AGA ATT-3′. The siRNA diluted solution was mixed with lipofectamine RNAiMAX to form an siRNA/lipofectamine RNAiMAX complex, which was added to the wells containing the cells and medium (no antibiotics). The cell culture plate was gently shaken and then incubated for 48 h at 37 °C in a CO_2_ incubator.

**Figure 2:**
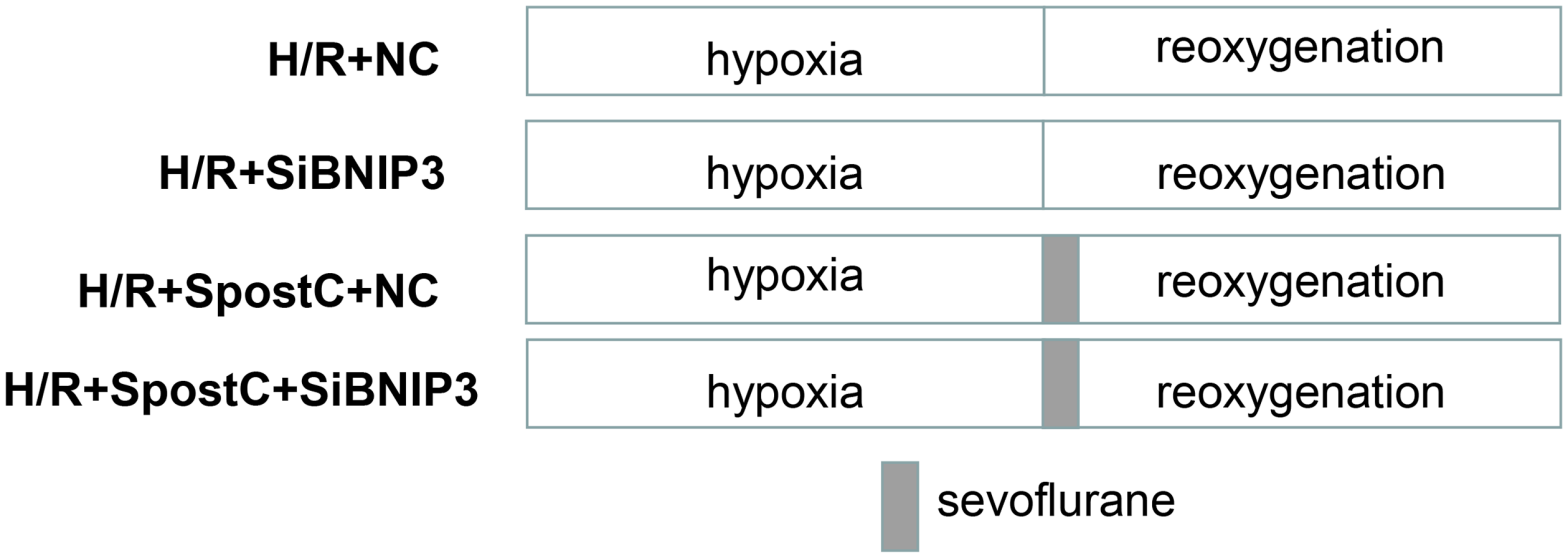
Experimental schemes of each group after silencing of BNIP3. H9C2 cells were randomly divided into hypoxia/reoxygenation + siRNA-NC (H/R+NC) group; hypoxia/reoxygenation + siRNA-BNIP3 (H/R+siBNIP3) group; hypoxia/reoxygenation + sevoflurane + siRNA-negative control (H/R+SPostC+NC) group; and hypoxia/reoxygenation + sevoflurane + siRNA-BNIP3 (H/R+SPostC+siBNIP3) group.

In the present study, siRNA-NC or siRNA-BNIP3 was transfected into cells, and after transfection, cells were exposed to hypoxic conditions for 3 h and reoxygenation for 3 h (SpostC: cells were exposed to 2.4% sevoflurane for 15 min at the beginning of reoxygenation after 3 h of hypoxia, and then incubated in a 5% CO_2_ cell incubator for 165 min). The treatments included hypoxia/reoxygenation + siRNA-NC (H/R+NC); hypoxia/reoxygenation + siRNA-BNIP3 (H/R+siBNIP3); hypoxia/reoxygenation + sevoflurane + siRNA-negative control (H/R+SPostC+NC); and hypoxia/reoxygenation + sevoflurane + siRNA-BNIP3 (H/R+SPostC+siBNIP3) (Fig. 2).

### Determination of cell viability

Cell viability was determined by the CCK-8 method. A total of 100 μl of 10% CCK-8 solution was added to each well of a 96-well plate, which was then incubated at 37 °C. After 2 h, the optical density values were measured at a wavelength of 450 nm using a plate reader.

### Determination of lactate dehydrogenase (LDH) content

According to the instructions of the LDH cytotoxicity test kit, cell supernatants of each well of the 96-well plate were collected and transferred to a new 96-well plate in each experimental group, and 60 µl of LDH test solution was added to each well. The plate was wrapped with foil and placed on a shaker to incubate for 30 min at room temperature. The absorbance values were then measured at 490 nm. The absorbance value was proportional to the LDH content.

### Apoptosis detection

Apoptosis was detected using an Annexin V-FITC/PI Assay Kit (Sigma, St. Louis, MO, USA). The cells were washed with precooled PBS and then resuspended in 100 μl of Annexin V binding solution to prepare single-cell suspensions. Then, 10 μl of Annexin V-FITC and 10 μl of PI staining solution were added and mixed gently, and the plate was placed at 4 °C for 15 min in the dark. An additional 500 μl of Annexin V binding solution was added. Flow cytometry detection was performed within 30 min.

### TEM analysis

A total of one ml of 2.5% glutaraldehyde was added to each group of H9C2 cells. Cells were fixed at 4 °C for 2 h, rinsed three times with precooled PBS and dehydrated with acetone. Next, 1% osmic acid was added at room temperature for 2 h. The cells were embedded in epoxy resin and dried, and ultrathin sectioning was performed after trimming. The cells were stained with uranyl acetate and lead citrate for 15 min and 5 min, respectively. The ultrastructural changes and autophagosomes in each group of H9C2 cells were observed under a transmission electron microscope. For autophagosome quantification, five micrographs, primary magnification × 20,000, were blindly taken from each group, and the total amount of autophagosomes was counted. Mitochondria were scored using a semi-quantitative analysis of FlaMeng as described previously, the higher the score, the more severe the mitochondrial damage (Jiang et al., 2016).

### Western blot analysis

H9C2 cells were lysed in ice–cold radioimmunoprecipitation assay lysis buffer, at 4 °C for 60 min and then the homogenate was incubated and centrifuged. The supernatant was collected, and the protein concentration was determined using the bicinchoninic acid protein assay kit according to the manufacturer’s protocol (Beyotime, Haimen, China). The supernatant was mixed with ×5 loading buffer and heated for 5 min at 100 °C, and then 30 micrograms of sample was subjected to electrophoresis using an SDS-PAGE gel system, transferred to a membrane and blocked at 37 °C for 2 h. Diluted primary antibodies to HIF-1α (1:200, abcam, ab1), BNIP3 (1:500, abcam, ab10433), and Beclin-1 (1:500, abcam, ab62557) were added, and the membrane was incubated overnight (4 °C). The membrane was washed with TBST solution and incubated with HRP-conjugated secondary antibody (1:10,000, sanggon, D110024) for 1 h at room temperature. Enhanced Chemiluminescence (ECL) was used for visualization and imaging. Gray value analysis of the target protein bands was performed using the Quantity One image analysis system.

### Statistical analysis

All experiments were independently repeated in the laboratory at least three times. SPSS 19.0 statistical software was used for the statistical analysis. Data are expressed as the mean ± standard deviation. Statistical significance was determined using a Student’s *t*-test for the comparison between two groups. Other comparisons were performed using a one-way ANOVA analysis with LSD post-hoc test for data with homogeneous variances, or Tamhane post-hoc test for data with non-homogeneous variances. A value of *P* < 0.05 was considered statistically significant. GraphPad Prism 5.0 was used to prepare graphs.

## Results

### SpostC alleviated cell injury induced by hypoxia and reoxygenation

**Figure 3:**
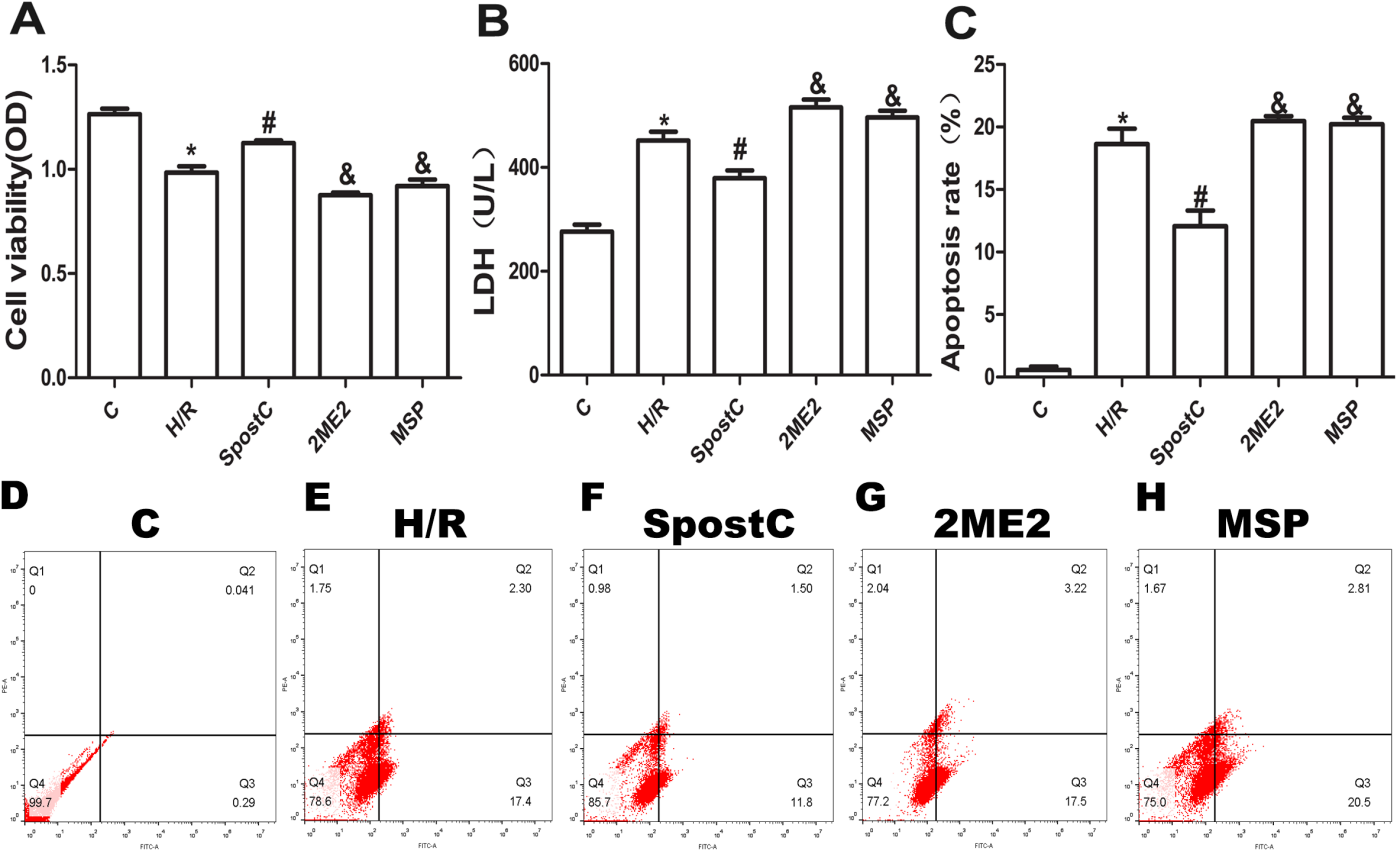
SpostC alleviated cell hypoxia-reoxygenation injury. (A) Cell viability: Compared to the H/R group, the SpostC group exhibited improved cell viability (*n* = 5/group from five independent experiments). (B) LDH activity: The LDH activity was reduced in the SpostC group compared to the H/R group (*n* = 5/group from five independent experiments). (C) Apoptosis rate: The apoptosis rate of the H/R group was significantly increased, while the apoptosis rate of the SpostC group was lower than that of the H/R group. The protective effects of SpostC were inhibited after administration of 2ME2 (*n* =3/group from three independent experiments). (D–H) Flow cytometry to measure apoptosis distribution graph. Data represent mean ± SD (*P < 0.05 vs C group, ^#^*P* < 0.05 vs H/R group, ^&^*P* < 0.05 vs SpostC group).

Cell viability, LDH activity, and apoptosis reflect the extent of cell damage. In this study, the cell viability of the H/R group was significantly lower than that of the control group (*P* < 0.05), while the cell viability of the SpostC group was increased compared to the H/R group (*P* < 0.05) (Fig. 3A). The LDH assay showed that compared to the control group, the LDH activity of the H/R group was increased (*P* < 0.05), while the LDH activity of the SpostC group was decreased compared to the H/R group (*P* < 0.05) (Fig. 3B). In addition, compared to the control group, the apoptotic rate of the H/R group was significantly increased (*P* < 0.05), while the apoptosis rate of the SpostC group was significantly lower than that of the H/R group (*P* < 0.05) (Fig. 3C).

### SpostC promoted mitochondrial autophagy via HIF-1α

**Figure 4:**
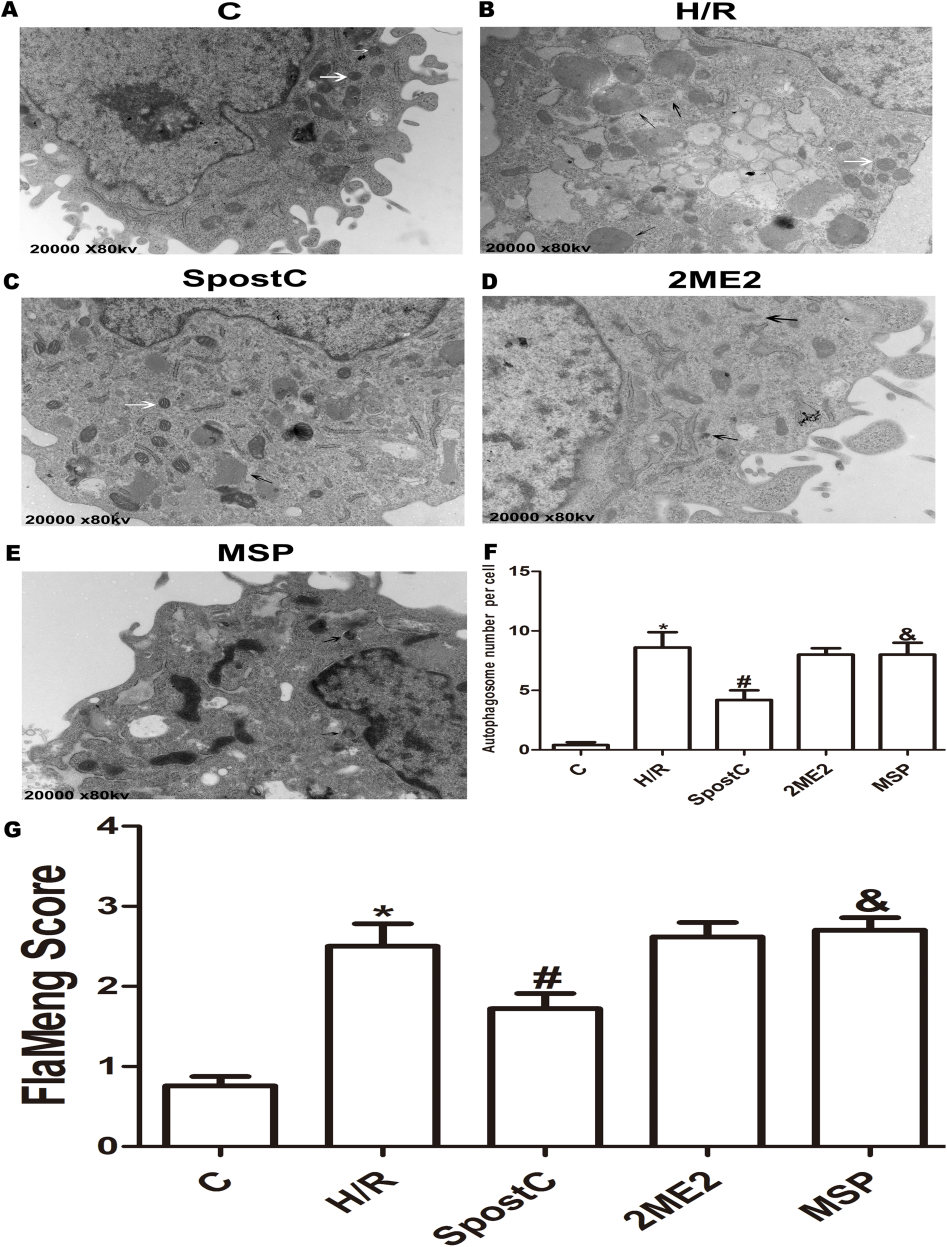
Mitochondrial structure and autophagosomes under electron microscopy. Autophagosomes are indicated by black arrows, mitochondria are indicated by white arrows. Flameng score indicating mitochondrial damage, the higher the score, the more severe the mitochondrial damage. Mitochondria in the H/R group were obviously swollen and many autophagosomes were present. The mitochondrial morphology in the SpostC group was largely normal, and the number of autophagosomes was reduced. (A) C group (B) H/R group (C) SpostC group (D) 2ME2 group (E) MSP group (F) Autophagosome number per cell (G) FlaMeng Score. Data represent mean ± SD (*n* = 5/group) (*P < 0.05 vs C group, ^#^*P* < 0.05 vs H/R group, ^&^*P* < 0.05 vs SpostC group).

**Figure 5:**
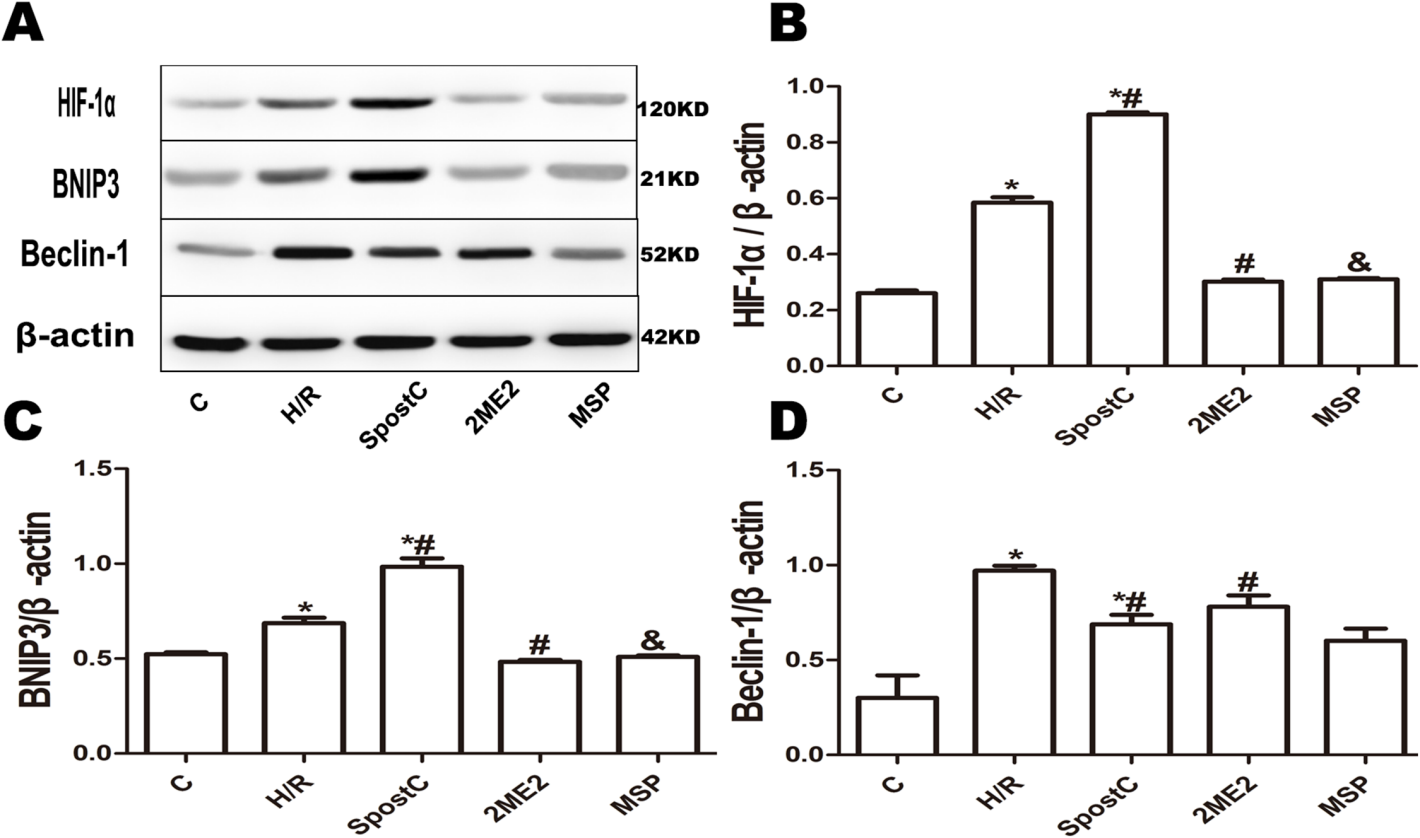
Effect of SpostC on HIF-1α, BNIP3 and Beclin-1 protein expression after H/R injury. (A) Western blot images of each group; (B) HIF-1α protein expression; (C) BNIP3 protein expression; (D) Beclin-1 protein expression; SpostC significantly upregulated HIF-1α and BNIP3 protein expression while decreasing Beclin-1 protein expression. After 2ME2 administration, the expression of HIF-1α and BNIP3 was downregulated. Data represent mean ± SD (*n* = 3/group) (*P < 0.05 vs C group, ^#^*P* < 0.05 vs H/R group, ^&^*P* < 0.05 vs SpostC group).

The morphological changes of mitochondria and autophagosomes were observed by transmission electron microscopy. The mitochondria in the H/R group were significantly swollen and contained a large number of autophagosomes compared to the control group; however, in the SpostC group, the mitochondrial morphology was normal, and the number of autophagosomes was significantly reduced (Figs. 4F and 4G). BCL2/ adenovirus E1B 19-kDa-interacting protein 3 is a key protein that regulates mitochondrial autophagy and a downstream target gene of HIF-1α. The results of this study showed that the expression levels of the HIF-1α and BNIP3 proteins in the SpostC group were significantly higher than those in the H/R group (Figs. 5B and 5C). However, the expression levels of the HIF-1α and BNIP3 proteins in the 2ME2 and MSP groups were significantly decreased after administration of 2ME2, an HIF-1α inhibitor (Figs. 5B and 5C), which was accompanied by mitochondrial swelling and accumulation of autophagosomes (Fig. 4), suggesting that SpostC promotes mitochondrial autophagy via HIF-1α.

### BNIP3 participated in mitochondrial autophagy promoted by SpostC

**Figure 6:**
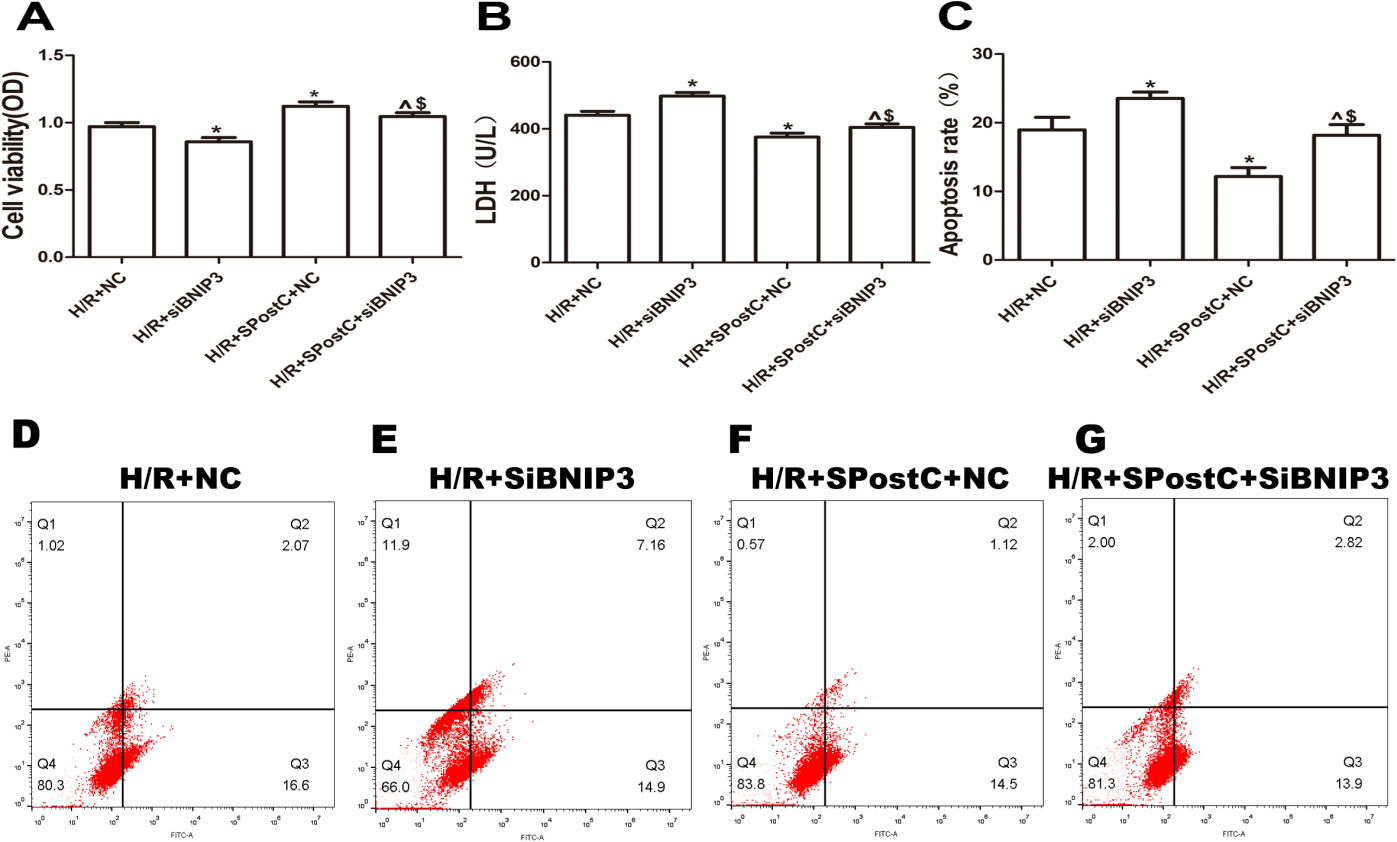
Silencing BNIP3 inhibited the protective effects of Spost C. (A) Cell viability: The cell viability of the H/R+SPostC+siRNA-BNIP3 group was lower than that of the H/R+SPostC+NC group (*n* = 5 from five independent experiments). (B) LDH activity: The H/R+SPostC+siRNA-BNIP3 group showed increased LDH activity compared to the H/R+SPostC+NC group (*n* = 5 from five independent experiments). (C) Apoptosis rate: The apoptosis rate of the H/R+SPostC+siRNA-BNIP3 group was significantly higher than that of the H/R+SPostC+NC group (*n* = 3/group from three independent experiments). (D–G) Flow cytometry to measure apoptosis distribution graph. Data represent mean ± SD (^*P* < 0.05 vs H/R+siBNIP3 group, *P < 0.05 vs H/R+NC group, ^$^*P* < 0.05 vs H/R+SpostC +NC group).

**Figure 7:**
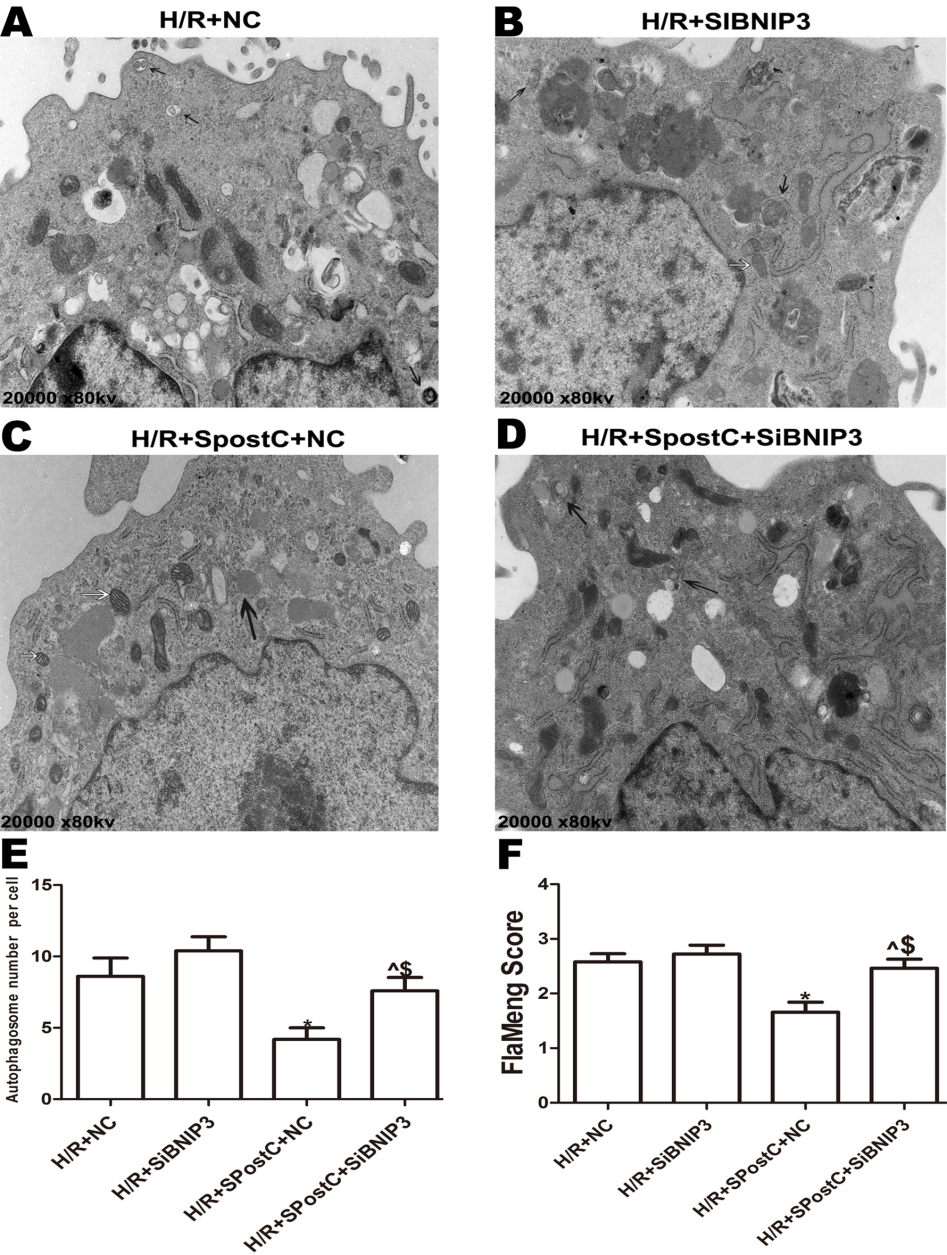
Mitochondrial structure and autophagosomes under electron microscopy after silencing of BNIP3. Autophagosomes are indicated by black arrows, mitochondria are indicated by white arrows. Flameng score indicating mitochondrial damage, the higher the score, the more severe the mitochondrial damage. Mitochondria were swollen in the BNIP3 silencing group, and autophagosomes were significantly accumulated. (A) H/R+NC group (B) H/R+siBNIP3 group (C) H/R+SpostC+NC group (D) H/R+SpostC+siBNIP3 group (E) Autophagosome number per cell (F) FlaMeng Score. Data represent mean ± SD (*n* = 5/group) (^*P* < 0.05 vs H/R+siBNIP3 group, *P < 0.05 vs H/R+NC group, ^$^*P* < 0.05 vs H/R+SpostC +NC group).

**Figure 8:**
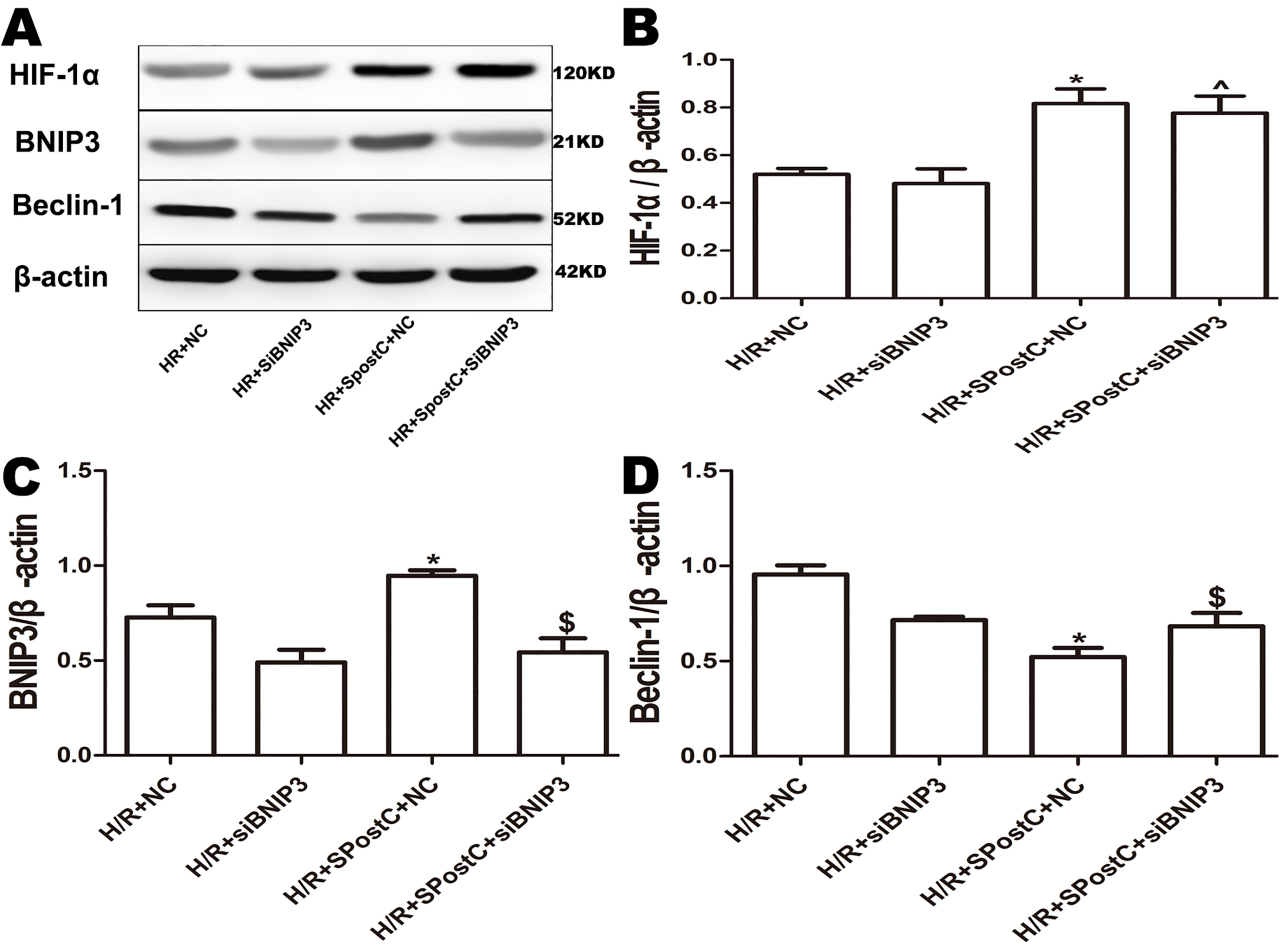
Expression of HIF-1α, BNIP3 and Beclin-1 protein in each group after silencing of BNIP3. (A) Western blot images of each group; (B) HIF-1α protein expression; (C) BNIP3 protein expression; (D) Beclin-1 protein expression; BNIP3 is a downstream target protein of HIF-1 to demonstrate that HIF-1/BNIP3 is involved in SPostC to promote mitochondrial autophagy by silencing BNIP3. Protein expression in the BNIP3 silencing groups was significantly downregulated. HIF-1α expression was not affected. Data represent mean ± SD (*n* = 3/group) (^*P* < 0.05 vs H/R+siBNIP3 group, *P < 0.05 vs H/R+NC group, ^$^*P* < 0.05 vs H/R+SpostC +NC group).

To confirm the role of BNIP3 in myocardial protection mediated by SpostC, the BNIP3 gene was silenced, which showed that cell viability was decreased (Fig. 6A), LDH activity was increased (Fig. 6B), the apoptotic rate was increased (Fig. 6C), mitochondrial damage was significant, and autophagosomes accumulated (Figs. 7E and 7F), BNIP3 expression was decreased (Fig. 8C) in the H/R+SPostC+siBNIP3 group and H/R+ siBNIP3 group compared to the H/R+SPostC+NC group. These results suggest that BNIP3 participates in mitochondrial autophagy promoted by SpostC through HIF-1α.

## Discussion

Autophagy is a self-regulated defensive adaptation process that is widely present in eukaryotic cells. This process involves autophagosomes encapsulating degenerated, damaged and aging intracellular proteins or organelles and transferring them to lysosomes for degradation. Macromolecules such as amino acids, nucleotides, and fatty acids are released, which provides raw materials for cell repair and reconstruction for normal turnover, renewal and recycling of the cytoplasmic components. This process plays an important regulatory role in maintaining intracellular homeostasis, cell growth, and development (Carames et al., 2010). Mitochondrial autophagy is a selective process that links mitochondrial degradation to autophagy and removes abnormal mitochondria through binding of autophagy proteins and specific mitochondrial autophagy proteins (Lemasters, 2005). Studies have shown that mitochondrial autophagy is an important mechanism of myocardial I/R injury (Jimenez, Kubli & Gustafsson, 2014). However, the role of autophagy in I/R injury is controversial (Gottlieb & Mentzer, 2013; Przyklenk et al., 2012). Autophagy protects the myocardium during myocardial ischemia, but autophagy overactivation during reperfusion has a damaging effect, which can lead to phagocytic cell death (Jang et al., 2013; Ma et al., 2012b). The specific mechanism of this action is not clear.

This study found that after myocardial cells underwent hypoxia-reoxygenation, cell viability was significantly reduced, LDH was increased, and the apoptosis rate was increased, accompanied by a significant decrease in mitochondria number and accumulation of a large number of autophagosomes. Additionally, expression of Beclin-1 protein, which reflects the degree of autophagy, was significantly elevated, indicating that Beclin-1 mediates the upregulation of autophagy during reperfusion (Matsui et al., 2007). Beclin-1 is the rate-limiting gene of autophagy initiation, participates in the regulation of autophagy and promotes the formation of autophagosomes, and their expression is also closely associated with autophagy activity (Fu, Cheng & Liu, 2013). The results of this study suggest that hypoxia-reoxygenation induces excessive autophagy, leading to cell death. One possible explanation might be that excessive autophagy not only engulfs damaged organelles but also consumes normal organelles, leading to damage. In addition, excessive autophagosome production leads to insufficient lysosomes that bind to autophagosomes and to incomplete degradation of autophagosomes, which in turn impairs autophagic flux, causing further damage to cells (Ma et al., 2015).

Myocardial I/R injury has always been a focus of research. Reducing myocardial cell death after reperfusion is also an enormous challenge (Hausenloy & Yellon, 2013). Studies have shown that SpostC can alleviate myocardial I/R injury. Our previous study also found that SpostC exerted myocardial protective effects by upregulating HIF-1α; however, the specific mechanism of this effect has not yet been elucidated. The present study found that SpostC could significantly improve cell viability, reduce LDH and decrease the apoptotic rate. The mitochondrial morphology in the SpostC group was normal under electron microscopy, and only a small number of autophagosomes were observed. These results suggest that SpostC might alleviate myocardial cell damage by promoting the mitochondrial autophagy pathway because the accumulation of autophagosomes may lead to the release of lysosomal proteases into the cytosol and ultimately induce cell death (Gottlieb & Mentzer, 2010). During reperfusion, autophagosomes accumulate due to blockade of transportation to lysosomes, and an increase in autophagic vacuoles may reflect a decrease in degradation activity. In the SpostC group, the expression of HIF-1α was upregulated, but the expression of Beclin-1 was decreased, indicating that the SpostC-induced promotion of mitochondrial autophagy via HIF-1α does not occur by increasing autophagosome formation but rather through accelerated autophagosome clearance. Treatment with HIF-1 inhibitors caused the protective effect of SpostC to disappear, which indicates that SpostC exerts its protective effects by upregulating HIF-1α to increase autophagosome clearance and inhibit autophagy activity or through accelerating autophagic flux.

Previous studies have shown that HIF-1 is a major factor in the regulation of cell survival under hypoxia, and BNIP3 is an important target gene downstream of HIF-1 (Cho et al., 2012). Under hypoxic conditions, HIF-1 can directly target the regulation of BNIP3 expression (Li et al., 2013). In addition, BNIP3 is a mitochondrial autophagy receptor and is considered to be an important signaling molecule for hypoxia-induced mitochondrial autophagy.

In this study, BNIP3 was upregulated in the SpostC group by HIF-1α expression, while Beclin-1 expression was downregulated. After 2ME2 administration, BNIP3 was downregulated along with HIF-1α expression, and Beclin-1 expression was upregulated, suggesting that BNIP3 is a downstream target gene of HIF-1α that participates in mitochondrial autophagy. In the pathway, BNIP3 and Beclin-1 may compete for binding to Bcl-2, and Beclin-1 is released to trigger autophagy (Jimenez, Kubli & Gustafsson, 2014). Beclin-1 can not only bind to Bcl-2 and Bcl-xl to regulate apoptosis but can also form a complex with PI3KC3 to play a key role in autophagy. It is an important protein for the exchange and coordination of the apoptosis and autophagy pathways (Kang et al., 2011). Further validating the role of BNIP3, the cardioprotective effects of SpostC were attenuated after silencing BNIP3, probably because BNIP3 can inhibit the fusion of damaged mitochondria, which allows the mitochondria to be easily eliminated (Gustafsson, 2011). BCL2/adenovirus E1B 19-kDa-interacting protein 3 silencing resulted in a decrease in BNIP3-dependent mitochondrial autophagy and apoptosis (Burton & Gibson, 2009). However, BNIP3 is a mitochondrial autophagy receptor that affects only mitochondrial autophagy and shows no effect on other types of autophagy (Feng et al., 2013).

In addition, Teixeira (Teixeira et al., 2015) and Onishi (Onishi et al., 2012) believe that pretreatment with isoflurane or sevoflurane before myocardial ischemia can inhibit the opening of mPTP to protect mitochondrial integrity and thus protect. However, I/R injury can cause different degrees of damage to mitochondria. Whether inhalation anesthetic preconditioning can protect against I/R injury by clearing damaged mitochondria is not mentioned. This study found that SpostC can promote the clearance of autophagosomes and avoid damaged mitochondria-derived ROS to damage the normal functional mitochondria, which is consistent with the conclusions of Yu et al. (2015). Furthermore, this study further confirmed that HIF-1/BNIP3 is involved in mediating the process of SpostC promote mitochondrial autophagy.

## Limitations

This study has some limitations. We demonstrated that SpostC promotes mitochondrial autophagy to alleviate hypoxia-reoxygenation injury in cardiomyocytes through the HIF-1/BNIP3 pathway only at cellular level, and this finding needs to be verified in the human body. Second, drugs were used to inhibit HIF-1α in this study instead of silencing or knockout techniques.

## Conclusions

In summary, SpostC can upregulate the expression of HIF-1α and promote the HIF-1/BNIP3 signaling pathway to regulate mitochondrial autophagy, thereby reducing autophagosome accumulation and myocardial hypoxia-reoxygenation injury.

## Supplemental Information

10.7717/peerj.7165/supp-1 Supplemental Information 1 Raw data for the indicators of each group. Raw data for the level of LDH release, cell viability, apoptosis rate, HIF-1a, BNIP3, Beclin-1. Number of autophagosomes, FlaMeng Score.

10.7717/peerj.7165/supp-2 Supplemental Information 2 Western blot images. Western blot images for the HIF-1α, BNIP3, and Beclin-1.

## Additional Information and Declarations

### Data Availability

The following information was supplied regarding data availability: The raw measurements are available in the Supplemental Files. The raw data shows LDH/CCK/Apoptosis rate of all group and each group of western bands includes HIF-1α, BNIP3, Beclin-1, number of autophagosomes.

## References

[ref-1] Bellot G, Garcia-Medina R, Gounon P, Chiche J, Roux D, Pouyssegur J, Mazure NM (2009). Hypoxia-induced autophagy is mediated through hypoxia-inducible factor induction of BNIP3 and BNIP3L via their BH3 domains. Molecular and Cellular Biology.

[ref-2] Burton TR, Gibson SB (2009). The role of Bcl-2 family member BNIP3 in cell death and disease: NIPping at the heels of cell death. Cell Death & Differentiation.

[ref-3] Cao J, Xie H, Sun Y, Zhu J, Ying M, Qiao S, Shao Q, Wu H, Wang C (2015). Sevoflurane post-conditioning reduces rat myocardial ischemia reperfusion injury through an increase in NOS and a decrease in phopshorylated NHE1 levels. International Journal of Molecular Medicine.

[ref-4] Caramés B, Taniguchi N, Otsuki S, Blanco FJ, Lotz M (2010). Autophagy is a protective mechanism in normal cartilage, and its aging-related loss is linked with cell death and osteoarthritis. Arthritis & Rheumatism.

[ref-5] Cho B, Choi SY, Park O-H, Sun W, Geum D (2012). Differential expression of BNIP family members of BH3-only proteins during the development and after axotomy in the rat. Molecules and Cells.

[ref-6] Feng D, Liu L, Zhu Y, Chen Q (2013). Molecular signaling toward mitophagy and its physiological significance. Experimental Cell Research.

[ref-7] Fu L-L, Cheng Y, Liu B (2013). Beclin-1: autophagic regulator and therapeutic target in cancer. International Journal of Biochemistry & Cell Biology.

[ref-8] Gottlieb RA, Mentzer RM (2010). Autophagy during cardiac stress: joys and frustrations of autophagy. Annual Review of Physiology.

[ref-9] Gottlieb RA, Mentzer RM (2013). Autophagy: an affair of the heart. Heart Failure Reviews.

[ref-10] Gui L, Liu B, Lv G (2016). Hypoxia induces autophagy in cardiomyocytes via a hypoxia-inducible factor 1-dependent mechanism. Experimental and Therapeutic Medicine.

[ref-11] Gustafsson AB (2011). Bnip3 as a dual regulator of mitochondrial turnover and cell death in the myocardium. Pediatric Cardiology.

[ref-12] Hausenloy DJ, Yellon DM (2013). Myocardial ischemia-reperfusion injury: a neglected therapeutic target. Journal of Clinical Investigation.

[ref-13] Jang BG, Choi BY, Kim JH, Kim M-J, Sohn M, Suh SW (2013). Impairment of autophagic flux promotes glucose reperfusion-induced neuro2A cell death after glucose deprivation. PLOS ONE.

[ref-14] Jiang J-J, Li C, Li H, Zhang L, Lin Z-H, Fu B-J, Zeng Y-M (2016). Sevoflurane postconditioning affects post-ischaemic myocardial mitochondrial ATP-sensitive potassium channel function and apoptosis in ageing rats. Clinical and Experimental Pharmacology and Physiology.

[ref-15] Jimenez RE, Kubli DA, Gustafsson AB (2014). Autophagy and mitophagy in the myocardium: therapeutic potential and concerns. British Journal of Pharmacology.

[ref-16] Kang R, Zeh HJ, Lotze MT, Tang D (2011). The Beclin 1 network regulates autophagy and apoptosis. Cell Death & Differentiation.

[ref-17] Lemasters JJ (2005). Selective mitochondrial autophagy, or mitophagy, as a targeted defense against oxidative stress, mitochondrial dysfunction, and aging. Rejuvenation Research.

[ref-18] Li C, Guan T, Chen X, Li W, Cai Q, Niu J, Xiao L, Kong J (2013). BNIP3 mediates pre-myelinating oligodendrocyte cell death in hypoxia and ischemia. Journal of Neurochemistry.

[ref-20] Ma X, Godar RJ, Liu H, Diwan A (2012a). Enhancing lysosome biogenesis attenuates BNIP3-induced cardiomyocyte death. Autophagy.

[ref-21] Ma X, Liu H, Foyil SR, Godar RJ, Weinheimer CJ, Hill JA, Diwan A (2012b). Impaired autophagosome clearance contributes to cardiomyocyte death in ischemia/reperfusion injury. Circulation.

[ref-19] Ma S, Wang Y, Chen Y, Cao F (2015). The role of the autophagy in myocardial ischemia/reperfusion injury. Biochimica et Biophysica Acta (BBA) - Molecular Basis of Disease.

[ref-22] Matsui Y, Takagi H, Qu X, Abdellatif M, Sakoda H, Asano T, Levine B, Sadoshima J (2007). Distinct roles of autophagy in the heart during ischemia and reperfusion: roles of AMP-activated protein kinase and Beclin 1 in mediating autophagy. Circulation Research.

[ref-23] Mizushima N, Komatsu M (2011). Autophagy: renovation of cells and tissues. Cell.

[ref-24] Obal D, Preckel B, Scharbatke H, Müllenheim J, Höterkes F, Thämer V, Schlack W (2001). One MAC of sevoflurane provides protection against reperfusion injury in the rat heart in vivo. British Journal of Anaesthesia.

[ref-25] Onishi A, Miyamae M, Kaneda K, Kotani J, Figueredo VM (2012). Direct evidence for inhibition of mitochondrial permeability transition pore opening by sevoflurane preconditioning in cardiomyocytes: comparison with cyclosporine A. European Journal of Pharmacology.

[ref-26] Przyklenk K, Dong Y, Undyala VV, Whittaker P (2012). Autophagy as a therapeutic target for ischaemia /reperfusion injury? Concepts, controversies, and challenges. Cardiovascular Research.

[ref-27] Qiao S-G, Sun Y, Sun B, Wang A, Qiu J, Hong L, An J-Z, Wang C, Zhang H-L (2018). Sevoflurane postconditioning protects against myocardial ischemia/reperfusion injury by restoring autophagic flux via an NO-dependent mechanism. Acta Pharmacologica Sinica.

[ref-28] Semenza GL (2011). Hypoxia-inducible factor 1: regulator of mitochondrial metabolism and mediator of ischemic preconditioning. Biochimica et Biophysica Acta (BBA) – Molecular Cell Research.

[ref-29] Teixeira G, Chiari P, Fauconnier J, Abrial M, Couture-Lepetit E, Harisseh R, Pillot B, Lacampagne A, Tourneur Y, Gharib A, Ovize M (2015). Involvement of cyclophilin D and calcium in isoflurane-induced preconditioning. Anesthesiology.

[ref-30] Wang X, Ma S, Qi G (2012). Effect of hypoxia-inducible factor 1-alpha on hypoxia/reoxygenation-induced apoptosis in primary neonatal rat cardiomyocytes. Biochemical and Biophysical Research Communications.

[ref-31] Wu J, Yu J, Xie P, Maimaitili Y, Wang J, Yang L, Ma H, Zhang X, Yang Y, Zheng H (2017). Sevoflurane postconditioning protects the myocardium against ischemia/reperfusion injury via activation of the JAK2–STAT3 pathway. PeerJ.

[ref-32] Yang L, Xie P, Wu J, Yu J, Yu T, Wang H, Wang J, Xia Z, Zheng H (2016). Sevoflurane postconditioning improves myocardial mitochondrial respiratory function and reduces myocardial ischemia-reperfusion injury by up-regulating HIF-1. American Journal of Translational Research.

[ref-33] Yu J, Wu J, Xie P, Maimaitili Y, Wang J, Xia Z, Gao F, Zhang X, Zheng H (2016). Sevoflurane postconditioning attenuates cardiomyocyte hypoxia/reoxygenation injury via restoring mitochondrial morphology. PeerJ.

[ref-34] Yu P, Zhang J, Yu S, Luo Z, Hua F, Yuan L, Zhou Z, Liu Q, Du X, Chen S, Zhang L, Xu G (2015). Protective effect of sevoflurane postconditioning against cardiac ischemia/reperfusion injury via ameliorating mitochondrial impairment, oxidative stress and rescuing autophagic clearance. PLOS ONE.

[ref-35] Zhang Y-L, Yao Y-T, Fang N-X, Zhou C-H, Gong J-S, Li L-H (2014). Restoration of autophagic flux in myocardial tissues is required for cardioprotection of sevoflurane postconditioning in rats. Acta Pharmacologica Sinica.

[ref-36] Zorov DB, Juhaszova M, Sollott SJ (2014). Mitochondrial reactive oxygen species (ROS) and ROS-induced ROS release. Physiological Reviews.

