# Supplementary figures and images for: Sevoflurane postconditioning alleviates hypoxia-reoxygenation injury of cardiomyocytes by promoting mitochondrial autophagy through the HIF-1/BNIP3 signaling pathway
Source: PeerJ. 2019 Jun 24;7:e7165. doi: 10.7717/peerj.7165 (PMC6596409; doi:10.7717/peerj.7165)

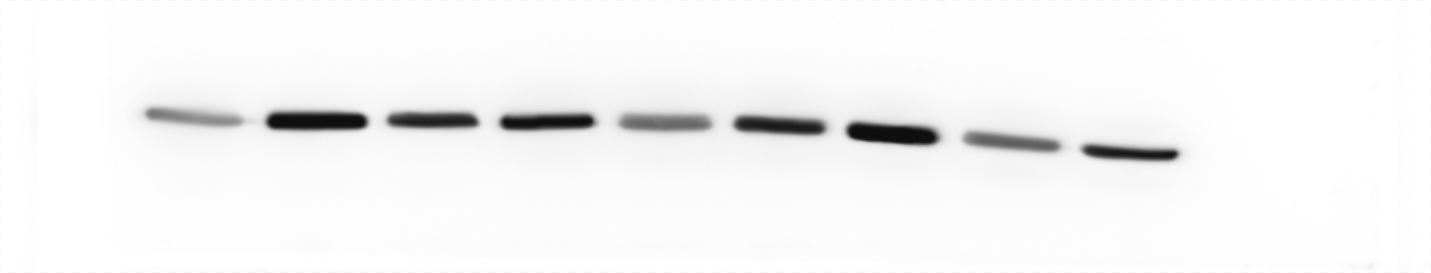

Supplement: Supplemental Information 2 — Western blot images for the HIF-1α, BNIP3, and Beclin-1. [file peerj-07-7165-s002.zip › Beclin-11C.2HR.3SPostC.42ME2.5MSP.6HR+NC.7HR+SiBNIP3.8HR+SPostC+NC.9 HR+ SPostC+ SiBNIP3.tif]

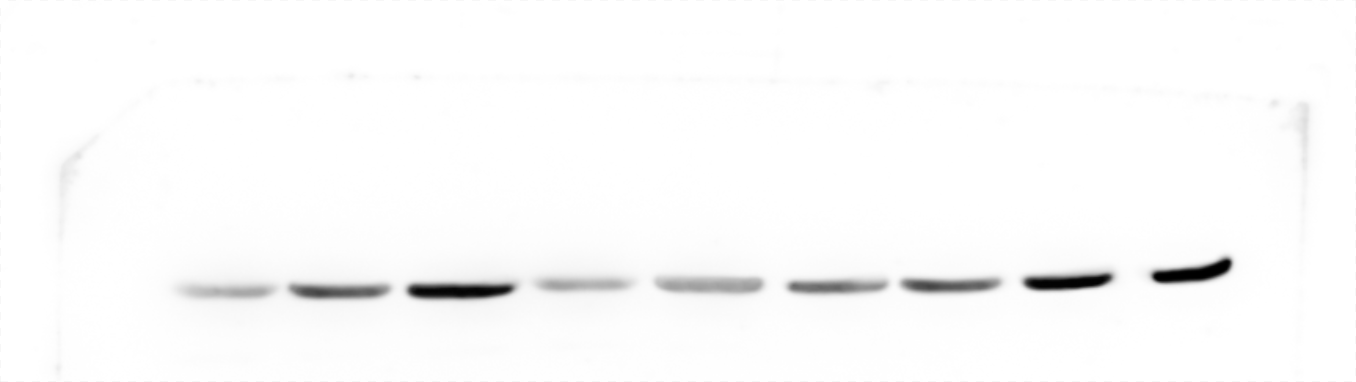

Supplement: Supplemental Information 2 — Western blot images for the HIF-1α, BNIP3, and Beclin-1. [file peerj-07-7165-s002.zip › HIF-1a┴1C.2HR.3SPostC.42ME2.5MSP.6HR+NC.7HR+SiBNIP3.8HR+SPostC+NC.9 HR+ SPostC+ SiBNIP3.tif]

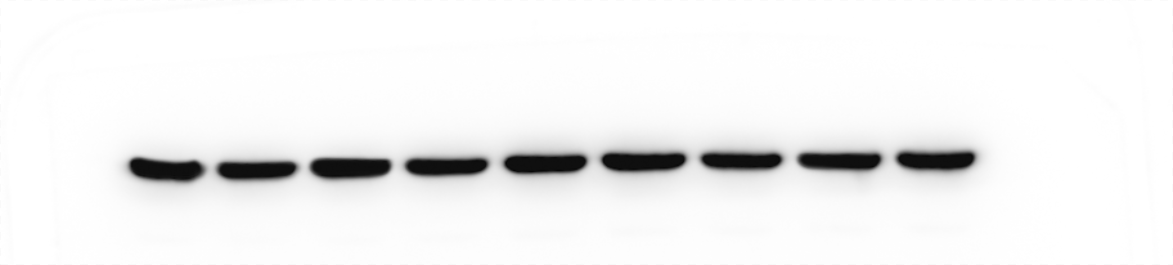

Supplement: Supplemental Information 2 — Western blot images for the HIF-1α, BNIP3, and Beclin-1. [file peerj-07-7165-s002.zip › actin.tif]

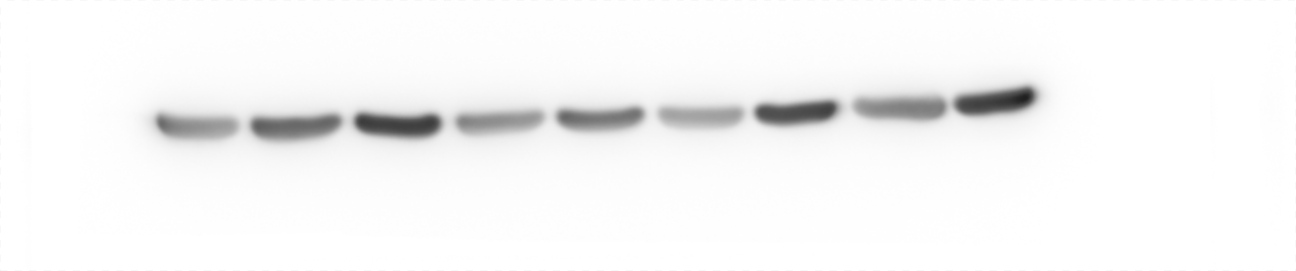

Supplement: Supplemental Information 2 — Western blot images for the HIF-1α, BNIP3, and Beclin-1. [file peerj-07-7165-s002.zip › BNIP31C.2HR.3SPostC.42ME2.5MSP.6HR+SiBNIP3.7HR+NC.8HR+ SPostC+ SiBNIP3.9 HR+SPostC+NC.tif]
